# Plasma IGFBP-2 Levels after Postoperative Combined Radiotherapy and Chemotherapy Predict Prognosis in Elderly Glioblastoma Patients

**DOI:** 10.1371/journal.pone.0093791

**Published:** 2014-04-01

**Authors:** Sheng Han, Lingxuan Meng, Shuai Han, Yunjie Wang, Anhua Wu

**Affiliations:** Department of Neurosurgery, The First Hospital of China Medical University, Shenyang, China; Beijing Tiantan Hospital, Capital Medical University, China

## Abstract

It has been found that preoperative plasma IGFBP-2 levels correlate with prognosis in glioma patients. The prognostic value of plasma IGFBP-2 after postoperative combined radiotherapy and chemotherapy in glioma patients is unknown. Plasma IGFBP-2 levels in 83 glioblastoma patients after postoperative radiotherapy plus chemotherapy were analyzed using an IGFBP-2 ELISA kit. We found that after standard therapy plasma IGFBP-2 levels significantly correlated with the patient's age (*R* = 0.738, *P*<0.001) and Karnofsky performance status (KPS, *R* = −0.633, *P*<0.05). Cox proportional hazards models were used to calculate hazard ratios (HRs) of death according to plasma IGFBP-2 levels adjusted for patient clinical characteristics. Plasma IGFBP-2 levels significantly correlated with overall survival in glioblastoma patients (multivariate HR = 1.035; 95% CI, 1.024–1.047; *P*<0.001). The effect of plasma IGFBP-2 levels on survival seemed to differ according to patients' age. Among patients older than 60, high plasma IGFBP-2 levels were associated with a significant increase in overall mortality (HR = 1.097; 95% CI, 1.055–1.140; *P*<0.001). In contrast, plasma IGFBP-2 levels conferred no significant effect on mortality among patients younger than 60. Elevated plasma IGFBP-2 levels after combined postoperative radiotherapy and chemotherapy in elderly glioblastoma patients correlate with poor KPS score and predicts poor prognosis.

## Introduction

Glioma is the most common primary tumor of the central nervous system, accounting for 80% of adult primary brain tumors [1]. Patients with the most advanced grade of glioma, glioblastoma multiforme (GBM), confront an extremely poor clinical outcome [2], [3], [4], [5], [6], [7], due to the highly proliferative and invasive nature of this tumor [8], [9], [10], [11]. The establishment of molecular markers would therefore be of considerable value in the identification of GBM tumor progression and the prediction of patient prognosis.

Previous studies have identified overexpression of insulin-like growth factor-binding protein 2 (IGFBP-2) as one of the most frequent molecular events in GBM [12], [13], [14]. However, IGFBP-2 is not highly expressed in all GBMs with poor prognosis, and IGFBP-2 expression in glioma tissues is not a suitable marker for prognosis prediction. Recently, preoperative plasma IGFBP-2 level has been associated with GBM patient prognosis [8]. IGFBP-2 expression is common to many tissues and organs, and is subject to regulation by numerous factors, including treatment methods, nutrition conditions and others, all of which significantly influence the IGFBP-2 levels of plasma [13], [15], [16]. Plasma IGFBP-2 levels may therefore change greatly after surgery and postoperative radiotherapy plus chemotherapy. Thus, in this study, we examined the prognostic value of plasma IGFBP-2 levels after postoperative combined radiotherapy and chemotherapy.

## Materials and Methods

### Study Population

We retrospectively examined blood samples and patient records from Chinese Glioma Genome Atlas (CGGA, http://www.cgcg.org.cn) for 83 patients who had been diagnosed with GBM. All patients received surgical resection from January 2007 to December 2009 and subsequently received similar radiotherapy and alkylating agent-based chemotherapy regimens according to Stupp protocol [17]. Surgical resection was performed by neurosurgeons who used similar operational techniques and principles. Samples were immediately snap-frozen in liquid nitrogen after resection. According to the 2007 World Health Organization (WHO) classification guidelines, the histological diagnosis was established and verified by two neuropathologists. Prior to the study, the percentage of tumor cells was evaluated using a hematoxylin and eosin–stained frozen section for each sample. Only samples with more than 80% tumor cells were selected for analysis. The patients had no other cancers or diseases such as acute infection, diabetes, and ischemia and received no other previous radiotherapy, chemotherapy, or corticosteroid therapy.

Tumor size was calculated based on preoperative MRI scans as follows: longest diameter × widest diameter × thickness (section thickness × the number of layers) ×1/2. Clinical data were retrospectively collected from medical records. The information on tumor resection for all patients examined was taken from medical charts. Extent of resection was defined as follows: (0) gross total resection (GTR), (1) partial removal with residual tumor <30%, and (2) residual tumor >30% or biopsy. The mean age of the patients was 56.7 years (range, 26–84 years). The median follow-up period for these patients was 13.8 months (range, 0.9 to 34.8 months). All the patients died of GBM at the end point of follow-up. Overall survival (OS) was defined as the interval between surgery and death from GBM. Patient clinical characteristics are summarized in **Table 1**. This study was approved by the institutional review board of the First Hospital of China Medical University, and written informed consent was obtained from every patient.

**Table 1:** Clinical and Molecular Characteristics of the 83 GBM Patients*.

Clinical or MolecularFeature	Cases		
	No.		%
**Total No. of patients**	83		
**Sex**			
Male	50		60.2
Female	33		39.8
**Age, years**			
Mean		56.7	
SD		17.8	
≥60	40		48.2
<60	43		51.8
**KPS**			
Mean		78.7	
SD		12.4	
**Tumor size, cm^3^**			
Mean		66.8	
SD		24.2	
**Extent of resection**			
Gross total resection	38		45.8%
Residual tumor <30%	20		24.1%
Residual tumor >30%	25		30.1%
**MGMT promoter**			
Methylated	25		30.1%
Unmethylated	58		69.9%
**Plasma IGFBP-2, ng/ml**			
Mean		637.0	
SD		52.3	
Median		627.5	

*KPS: Karnofsky performance status; IGFBP-2: Insulin-like growth factor binding protein 2; MGMT: O(6)-methylguanine-DNA-methyltransferase.

### Plasma Sample Collection

Blood was taken from GBM patients the day after postoperative radiotherapy plus chemotherapy. Blood samples were collected in the morning after an overnight fast and drawn into tubes with EDTA anticoagulant. The blood was centrifuged at 3,500 rpm for 10 min to recover plasma. Then, the upper-layer plasma was collected, aliquoted into Eppendorf tubes and then recentrifuged at 10,000 rpm for 3 min at 4°C to further remove white cells and debris. The extracted plasma was stored at −80°C until analysis.

### ELISA Assays

The IGFBP-2 ELISA kit was purchased from RapidBio Lab, Calabasas, CA, USA. IGFBP-2 levels in plasma were measured according to the manufacturer's instructions. Examination was repeated three times for each sample, and an average concentration of plasma IGFBP-2 was calculated and used for statistical analysis.

### MGMT promoter methylation analysis by MSP

Methylation-specific PCR (MSP) was performed as we previously described [10] to detect O(6)-methylguanine-DNA-methyltransferase (MGMT) promoter methylation. Briefly, tissue samples were lysed with 490 μL lysis buffer containing 20 mM Tris-Cl (pH 8.0), 5 mM EDTA (pH 8.0), 400 mM NaCl and 1% (w/v) SDS, and digested with 10 μL proteinase K at 10 mg/mL at 37°C for 12 h. Genomic DNA was purified from the lysate by phenol/chloroform extraction. One microgram of DNA was denatured by NaOH and modified by sodium bisulfite. MSP was performed using primer sequences for MGMT as follows: 5′-TTT GTG TTT TGA TGT TTG TAG GTT TTT GT-3′ (forward) and 5′-AAC TCC ACA CTC TTC CAA AAA CAA AAC A-3′ (reverse) for the unmethylated reaction; and 5′-TTT CGA CGT TCG TAG GTT TTC GC-3′ (forward) and 5′-GCA CTC TTC CGA AAA CGA AAC G-3′ (reverse) for the methylated reaction. Each PCR reaction (10 μL) was loaded onto nondenaturing 6% polyacrylamide gels, stained with ethidium bromide, and visualized under UV illumination. The PCR reaction was repeated at least three times.

### Survival Analysis and Statistical Analysis

Cox regression analysis was used to calculate hazard ratios (HRs) of death according to plasma IGFBP-2 level after postoperative radiotherapy plus chemotherapy, unadjusted and adjusted for age, sex, Karnofsky performance status (KPS, after postoperative combined radiotherapy and chemotherapy), tumor size, extent of resection and MGMT promoter methylation status. To adjust for potential confounding, age, KPS, tumor size and plasma IGFBP-2 levels were used as continuous variables and all of the other covariates were used as categorical variables (**Table 1**). Kaplan-Meier analysis was used to describe the distribution of overall survival time, and the log-rank test was performed.

Correlations between plasma IGFBP-2 level, age and KPS were analyzed by Pearson correlation analysis (SPSS 13.0). The student's *t*-test and ANOVA were used to determine statistical significance. Each experiment was done in triplicate. All data are presented as the mean ± standard error of three independent experiments. A two-tailed *P*-value of <0.05 was regarded as significant.

## Results

### Plasma IGFBP-2 Level after combined Treatment Correlated with Age and KPS in GBM Patients

**Figure 1:**
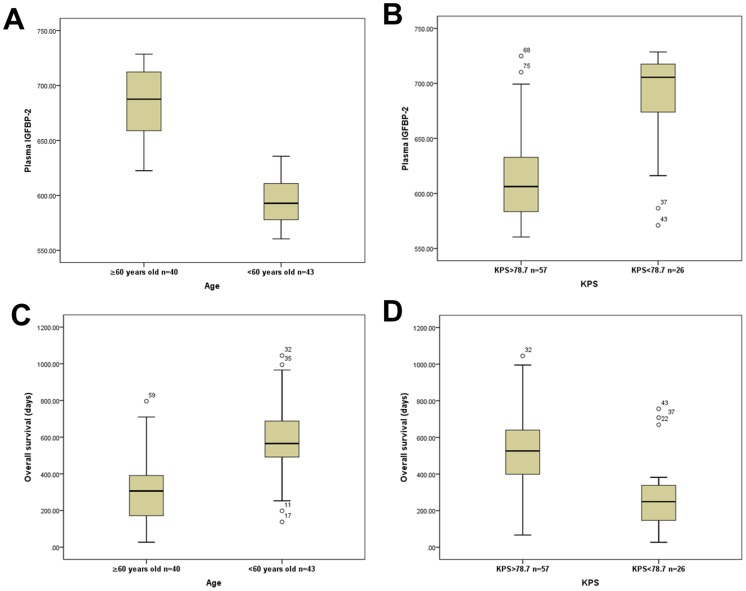
Correlation of plasma IGFBP-2 level with age and KPS in GBM patients after postoperative radiotherapy plus chemotherapy. (A, B) Box plots demonstrate that older age (≥60 years) or lower KPS values (<78.7) correlate with markedly higher plasma IGFBP-2 level (ng/ml) respectively (*P*<0.05). (C, D) Box plots show that older age (≥60 years) or lower KPS values (<78.7) are associated with significantly shorter overall survival (*P*<0.05).

We measured plasma IGFBP-2 levels of the 83 GBM patients after postoperative combined radiotherapy and chemotherapy using ELISA assay. The mean plasma IGFBP-2 level was 637.0±52.3 ng/ml (range, 560.4–728.6 ng/ml). After standard therapy, plasma IGFBP-2 level significantly correlated with the patient's age (*R* = 0.738, *P*<0.001). As shown in **Fig 1A**, we found that IGFBP-2 levels in plasma were markedly higher in patients older than 60 (mean±SD: 683.3±33.3 ng/ml) than in patients younger than 60 (593.9±20.8 ng/ml; *P*<0.05). Moreover, OS was substantially decreased in elderly patients (mean ±SD: 304.2±171.3 days) compared to younger patients (582.1±188.1 days; *P*<0.05; **Fig 1C**).

In addition, after postoperative radiotherapy and chemotherapy, plasma IGFBP-2 level also negatively correlated with patient KPS (*R* = −0.633, *P*<0.05, median KPS = 78.7). Plasma IGFBP-2 levels were remarkably higher in patients with KPS values less than 78.7 (mean ±SD: 689.4±41.4 ng/ml) than in patients with KPS values greater than 78.7 (613.1±37.8 ng/ml; *P*<0.05; **Fig 1B**). OS was markedly decreased in patients with KPS values less than 78.7 (mean±SD: 276.5±190.5 days) relative to patients with KPS values greater than 78.7 (526.4±198.6 days; *P*<0.001; **Fig 1D**).

**Figure 2:**
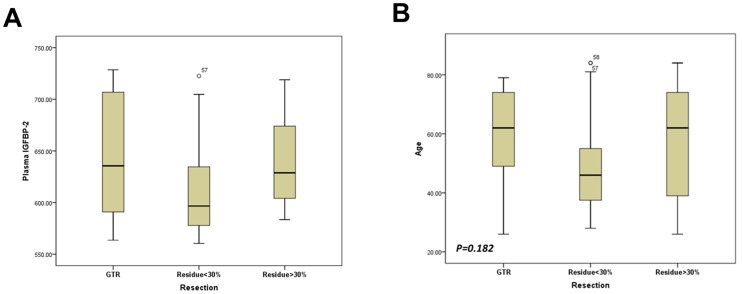
Correlation between clinical variables in the 83 GBM patients. (A) After combined therapy plasma IGFBP-2 did not correlate with the extent of resection. (B) The extent of resection was not associated with age.

We found that, after postoperative radiotherapy plus chemotherapy, plasma IGFBP-2 levels did not vary significantly with sex, preoperative tumor size and MGMT promoter methylation status. Moreover, plasma IGFBP-2 levels seem to be not associated with the extent of resection, since plasma IGFBP-2 levels were similar between patients underwent GTR and patients with residual tumor >30% (mean±SD, 646.4±57.0 ng/ml and 643.3±43.7 ng/ml respectively, **Fig 2A**). The extent of resection was mainly determined by considerations including functional preservation and general state of the patients. In this series of patients, extent of resection did not significantly correlate with patients' age (**Fig 2B**).

### Plasma IGFBP-2 Level after combined Treatment Correlated with the Prognosis in GBM patients

**Figure 3:**
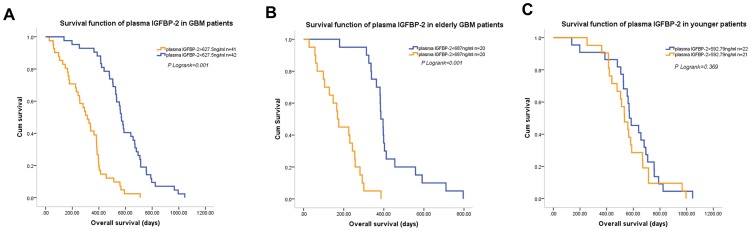
Kaplan-Meier curves for overall survival (OS) in GBM according to plasma IGFBP-2 levels after postperative radiotherapy plus chemotherapy. (A) GBM patients with high plasma IGFBP-2 (>627.5 ng/ml) had a significantly worse OS than did GBM patients with low plasma IGFBP-2 (<627.5 ng/ml; P<0.001).(B) In GBM patients older than 60, high plasma IGFBP-2 (>687 ng/ml) was associated with significantly shorter OS (P<0.001). (C) In GBM patients younger than 60, plasma IGFBP-2 levels were not associated with OS.

Next, we examined the survival function of plasma IGFBP-2 levels after combined treatment in GBM patients. Since the median plasma IGFBP-2 level in the 83 GBM patients was 627.5 ng/ml, we used a level of 627.5 ng/ml as the cutoff point to divide the GBM patients into two equal subgroups. Plasma IGFBP-2 level significantly discriminated the survival of the two GBM subgroups, with a mean OS of 303.6±24.9 days (median OS, 313 days; 95% CI, 244.0–382.0 days) for patients with plasma IGFBP-2>627.5 ng/ml versus a mean OS of 589.3±29.8 days (median OS, 568 days; 95% CI, 536.2–599.8 days, **Fig 3A**) for patients with plasma IGFBP-2<627.5 ng/ml. Univariate and multivariate Cox regression analysis showed that, plasma IGFBP-2 level was an independent predictor for OS of GBM patients (multivariate HR = 1.035, 95% CI 1.024–1.047, *P*<0.001, **Table 2**). In this study, multivariate analysis showed that KPS, extent of resection and MGMT promoter methylation status were also associated with OS in GBM. While the OS of patients underwent GTR and OS of patients with residual tumor <30% were similar (mean OS, 481.0±46.0 days and 501.0±33.1 days respectively), patients with residual tumor >30% significantly experienced a shorter OS (mean OS, 356.0±29.6 days).

**Table 2:** Univariate and Multivariate Cox Hazards Analysis in the 83 GBM Patients.

Covariate	Univariate			Multivariate		
	Hazard ratio	95% CI	*P*	Hazard ratio	95% CI	*P*
Age	1.028	1.013–1.042	<0.001	0.990	0.971–1.010	0.344
Sex	0.812	0.516–1.277	0.368	1.384	0.843–2.273	0.199
KPS	0.957	0.935–0.980	<0.001	0.971	0.944–0.999	0.044
Tumor size	0.997	0.989–1.006	0.539	1.005	0.996–1.015	0.265
Extent of resection	1.602	1.201–2.136	0.001	1.656	1.226–2.238	0.001
MGMT	0.455	0.276–0.750	0.002	0.570	0.335–0.969	0.038
Plasma IGFBP-2	1.032	1.024–1.040	<0.001	1.035	1.024–1.047	<0.001

*KPS: Karnofsky performance status; IGFBP-2: Insulin-like growth factor binding protein 2;

MGMT: MGMT promoter methylation status.

### The Prognostic Effect of Plasma IGFBP-2 Levels in Different Age Groups

Given the relationship between patient age and plasma IGFBP-2 levels after postoperative radiotherapy and chemotherapy, we next examined the prognostic effect of plasma IGFBP-2 levels in different age groups. As shown in **Fig 3B**, in patients older than 60, the median plasma IGFBP-2 level was 687 ng/ml, which was used as the cutoff point. The plasma IGFBP-2 level effectively discriminated between the survival of the two subgroups, with a mean OS of 183.3±21.9 days (median OS, 169.0 days; 95% CI, 147.1–190.9 days) for patients with plasma IGFBP-2>687 ng/ml versus a mean OS of 425.1±31.5 days (median OS, 385.0 days; 95% CI, 356.5–413.5 days) for patients with plasma IGFBP-2<687 ng/ml. Elevated plasma IGFBP-2 level was associated with a significant increase in overall mortality (multivariate HR = 1.097; 95% CI, 1.055 to 1.140, *P*<0.001; **Table 3**). In contrast, plasma IGFBP-2 levels conferred no significant effect on patient outcome among patients younger than 60 (multivariate HR = 1.001; 95% CI 0.985–1.018, *P* = 0.869; **Fig 3C**, **Table 4**). As shown in **Table 3** and **Table 4**, in this study, poor KPS after postoperative radiotherapy plus chemotherapy correlated with a marked increase in the overall mortality in the elderly group and residual tumor >30% predicted poor prognosis in younger patients.

**Table 3:** Univariate and Multivariate Cox Hazards Analysis in Elderly GBM Patients.

Covariate	Univariate			Multivariate		
	Hazard ratio	95% CI	*P*	Hazard ratio	95% CI	*P*
Age	0.984	0.939–1.032	0.512	0.976	0.925–1.029	0.371
Sex	0.734	0.368–1.466	0.381	1.184	0.532–2.634	0.679
KPS	0.780	0.716–0.850	<0.001	0.883	0.802–0.972	0.011
Tumor size	0.997	0.985–1.009	0.604	0.986	0.968–1.005	0.139
Extent of resection	0.976	0.699–1.364	0.888	1.164	0.750–1.806	0.499
MGMT	0.465	0.221–0.976	0.043	0.203	0.067–0.616	0.005
Plasma IGFBP-2	1.064	1.044–1.085	<0.001	1.097	1.055–1.140	<0.001

*KPS: Karnofsky performance status; IGFBP-2: Insulin-like growth factor binding protein 2; MGMT: MGMT promoter methylation status.

**Table 4:** Univariate and Multivariate Cox Hazards Analysis in Younger GBM Patients.

Covariate	Univariate			Multivariate		
	Hazard ratio	95% CI	*P*	Hazard ratio	95% CI	*P*
Age	0.983	0.955–1.012	0.260	0.987	0.955–1.021	0.450
Sex	0.538	0.284–1.018	0.057	1.055	0.515–2.159	0.884
KPS	1.162	1.074–1.257	<0.001	1.034	0.951–1.124	0.434
Tumor size	0.998	0.986–1.010	0.728	1.002	0.987–1.017	0.816
Extent of resection	6.110	3.344–11.165	<0.001	5.641	2.638–12.06	<0.001
MGMT	0.358	0.177–0.726	0.004	0.335	0.142–0.791	0.013
Plasma IGFBP-2	1.003	0.989–1.018	0.641	1.001	0.985–1.018	0.869

*KPS: Karnofsky performance status; IGFBP-2: Insulin-like growth factor binding protein 2; MGMT: MGMT promoter methylation status.

We further examined the influence of elevated plasma IGFBP-2 levels on survival across strata of the extent of resection or MGMT promoter methylation status in the two age groups. The increased risk of death was apparent in all subgroups among patients older than 60. However, plasma IGFBP-2 levels were not associated with OS in any subgroup among patients younger than 60.

### The Prognostic Effect of Plasma IGFBP-2 Levels in Different KPS Groups

**Figure 4:**
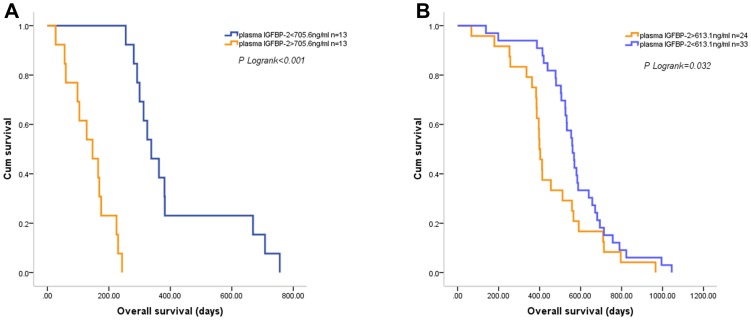
The prognostic effect of plasma IGFBP-2 levels in different KPS groups. Both in patients with KPS value less than 78.6 (A) and in patients with KPS value greater than 78.6 (B), elevated plasma IGFBP-2 levels after combined therapy were associated with shorter OS.

We next examined the prognostic effect of plasma IGFBP-2 levels in different KPS groups. The mean KPS value of the patients was 78.7, which was used to divide the two subgroups. In patients with KPS value less than 78.7, elevated plasma IGFBP-2 level was associated with a significant increase in overall mortality (multivariate HR = 1.488; 95% CI, 1.160 to1.910, *P* = 0.002). In patients with KPS value greater than 78.7, elevated plasma IGFBP-2 level also correlated with a marked increase in overall mortality (multivariate HR = 1.026; 95% CI, 1.012 to 1.040, *P*<0.001). Using plasma IGFBP-2 levels of 705.6 ng/ml (median) and 613.1 ng/ml (mean) respectively, the survival of the two subgroups can be effectively discriminated (**Fig 4**).

## Discussion

Regardless of aggressive approaches such as surgery, combined chemotherapy and radiotherapy, the outcome of patients with GBM remains poor [17]. Clinical experience has shown, however, that different patients experience distinct prognoses in response to the same treatment, and the development of biomarkers, particularly serum markers, that reflect the efficacy of treatment and the prognosis in GBM patients is an important goal.

While IGFBP-2 is a known potent modulator of the mitogenic effects of IGFs [18], IGF-independent actions of IGFBP-2 have been recently recognized, suggesting that IGFBP-2 is a regulator of tumor growth and invasiveness in its own right [19], [20]. Serum IGFBP-2 levels and tumor IGFBP-2 expression are dramatically elevated in a variety of human malignancies including GBM [14], [21], [22], and it has been reported that preoperative plasma IGFBP-2 levels are correlated with recurrence and disease-free survival in patients with GBM [8]. Given that plasma IGFBP-2 level is under dynamic changes in response to factors such as a patient's systemic state and specific treatment regimens, however, determining the prognostic value of plasma IGFBP-2 level after postoperative radiotherapy plus chemotherapy has substantial potential value.

Previous studies demonstrated that although IGFBP-2 expression was remarkably up-regulated in GBMs, the prognostic value of its expression in GBM tissue was uncertain [23], [24]. We found that after surgery and combined radiotherapy plus chemotherapy, plasma IGFBP-2 levels remained significantly high in certain patients. While it is unclear why plasma IGFBP2 remains elevated in these patients, we can surmise that the primary source of plasma IGFBP-2 in these patients may not be the tumors. For one thing, plasma IGFBP-2 levels were not associated with the extent of resection and residual tumor after surgery. For another thing, plasma IGFBP-2 levels were remarkably high in some radiologically tumor-free patients after combined therapy. Consistent with this notion, Lin et al reported that in patients with GBM, there was no correlation between plasma IGFBP-2 level and tumor IGFBP-2 expression as well as tumor size [8]. Collectively, these results indicate that plasma IGFBP-2 in GBM patients has other origins besides the tumor. However, we cannot accurately assess the residual tumor volume after postoperative radiotherapy plus chemotherapy, since in some cases it is very difficult to discriminate between progression and pseudoprogression. Also, we cannot measure the IGFBP-2 expression level in residual tumor after combined treatment. Thus, the origin of plasma IGFBP-2 after standard therapy needs to be further explored.

We demonstrated that elevated plasma IGFBP-2 levels persist in elderly GBM patients after postoperative radiotherapy plus chemotherapy, and that high plasma IGFBP-2 is associated with a marked increase in overall mortality. In patients younger than 60 years, however, the correlation between plasma IGFBP-2 levels and prognosis was insignificant. Plasma IGFBP-2 therefore appears to play a more important role in the prognosis of elderly patients.

Two factors might explain why elevated plasma IGFBP-2 leads to poor prognosis in elderly patients. Firstly, the plasma IGFBP-2 level may reflect the systemic status of the patient. It has been reported that high serum IGFBP-2 concentrations are a powerful indicator of overall poor physical functional status in elderly men [16], [25]. Similarly, in this study, we showed that higher plasma IGFBP-2 levels were correlated with lower KPS, a commonly used indicator of overall systemic status, and a well-known predictor for prognosis. Secondly, elevated plasma IGFBP-2 may affect residual tumor tissue and promote tumor growth. It has been reported that exogenous IGFBP-2 promotes proliferation and/or invasion of many cancer cells, including epithelial ovarian carcinoma cells [19], breast cancer cells [26] and neuroblastoma cells [27]. And in the future study, we will test the effect of exogenous IGFBP-2 on the growth of GBM cells.

Levels of circulating IGFBP-2 are highly regulated by metabolic, hormonal and developmental factors, and can be up-regulated by various stimuli, including fasting stress and other factors [28]. Unfortunately, during treatment, GBM patients frequently experience such conditions: for example, vomiting caused by chemotherapy and nervousness can elevate plasma IGFBP-2 level. Our study therefore emphasizes the importance of therapeutic intervention for GBM patients to decrease plasma IGFBP-2 levels. However, the mechanisms by which IGFBP-2 affects prognosis of GBM patients may be complicated, since plasma IGFBP-2 levels have been related to many diseases [29], [30], [31] other than solid tumors.

The lack of serial dynamic plasma IGFBP-2 levels is a limitation of our study, as is the small number of cases. Our data indicate, however, that elevated plasma IGFBP-2 levels after postoperative radiotherapy plus chemotherapy in elderly GBM patients is a predictor of poor prognosis, and that strategies to reduce plasma IGFBP-2 levels may be a suitable treatment approach in GBM patients. A greater understanding of the correlation between plasma IGFBP-2 level and treatment efficacy will be of considerable clinical significance.
